# CT target scanning in the diagnosis of solid pseudopapillary tumor of the pancreas

**DOI:** 10.1186/s12880-023-01175-3

**Published:** 2023-12-15

**Authors:** Hai-tao Wang, Yu-tao Wang, Zhi-hai Yu, Can Tu, Bin Lu, Liang Yu, Di Feng, Tie-gong Wang

**Affiliations:** 1 Department of Radiology, The First Affiliated Hospital of Ningbo University, No.247 Renmin Road, Jiangbei District, Ningbo, 315020 China; 2 Department of Radiology, Ningbo Ninth Hospital, No.68 Xiangbei Road, Jiangbei District, Ningbo, 315020 China; 3 Department of Radiology, Changhai Hostipal, Naval Medical University, No.168 Changhai Road, Yangpu District, Shanghai, 200433 China

**Keywords:** Diagnosis, Solid pseudopapillary tumor of the pancreas, X-ray computed tomography

## Abstract

**Objective:**

To discuss the value of computed tomography (CT) iterative reconstruction technique combined with target scanning in the diagnosis of solid pseudopapillary tumor of the pancreas (SPTP).

**Methods:**

The clinical information and CT examination data of 27 patients with SPTP were retrospectively analyzed, and the general condition and CT performance of the patients were observed. The CT image reconstruction algorithm of all patients used iterative reconstruction technique combined with the application of target scanning technique.

**Results:**

A total of 27 patients were included in this study, including 6 males and 21 females, aged 14–72 years with a mean age of 39.6 ± 13.6 years. SPTP was more common in young and middle-aged females, with a low level of tumor markers, dominated by cystic-solid tumors. The combination of CT iterative reconstruction technology and targeted scanning revealed the following: the capsule of SPTP was clear and complete, where calcifications were visible, solid components were progressively enhanced, and rare pancreatic and bile duct dilation was seen. Tumors were cystic-solid in 18 of 27 patients with SPTP, of which the solid components showed isodensity or slightly low-density, with calcifications. The solid components and cyst walls were mildly enhanced during the arterial phase, and were progressively enhanced during the parenchymal phase, portal vein phase, and delayed phase, with their enhancement degree lower than that of normal pancreatic parenchyma, and pancreatic and bile duct dilation was rare. There were no statistical differences in tumor location, morphology, growth pattern, integrity of capsule, cystic or solid, calcifications, and enhancement features between the male group and the female group (*P* > 0.05).

**Conclusion:**

The iterative reconstruction combined with target scanning clearly displayed the CT features of tumors, helping the diagnosis and clinical treatment of the disease.

## Introduction

Solid pseudopapillary tumor of the pancreas (SPTP), a rare type of exocrine pancreatic tumor, represents 0.9–2.7% [1] of all pancreatic tumors and is most often diagnosed in young women [2]. SPTP, as a tumor with malignant potential, [3] requires surgery. The combination of computed tomography (CT) iterative reconstruction technique, which improves the image quality while reducing the radiation dose, and target scanning, which has merits such as a small field of view (FOV) and high definition of focal details, helps to improve the diagnostic efficiency of this disease [4, 5] .The present use of target scanning focuses mainly on the diagnosis of pulmonary nodules, with only a few available reports on studies of pancreatic tumors. In this study, we retrospectively analyzed the clinical and imaging data of 27 patients with SPTP patients to explore the utility of the CT iterative reconstruction technique combined with target scanning in the diagnosis of SPTP.

## Materials and methods

### General data

In this paper, we describe a retrospective cross-sectional study that we conducted. We collected the clinicopathological data of 27 patients who underwent pancreatic CT enhanced examination in the First Affiliated Hospital of Naval Medical University and the First Affiliated Hospital of Ningbo University from October 2016 to May 2018 and were diagnosed with SPTP by surgical pathology. The samples consisted of 6 males and 21 females aged 14–72 years, with an average age of (39.6 ± 13.6) years. The main clinical symptoms included abnormal pain in 10 cases, low back pain in 3 cases, nausea and vomiting in 1 case, and pancreatic lesion incidentally found during physical examination, while there were no obvious symptoms in 13 cases. Tumor marker investigations showed that alpha-fetoprotein (AFP), carbohydrate antigen 19 − 9 (CA199), and carcinoembryonic antigen (CEA) were all negative. This study was approved by the Ethics Committee of our hospital. Patients and their family members signed the informed consent for CT enhanced examination and surgery.

### Inclusion and exclusion criteria

Inclusion criteria: (1) Patients who underwent surgical excision and were diagnosed with SPTP by pathology examination. (2) Patients who underwent TOSHIBA’s 320-slice CT enhanced examination before the surgery. (3) Patients whose CT examination image was of good quality and showed no obvious motion artifacts. (4) Patients who did not undergo anti-tumor treatment or puncture biopsy before the surgery.

Exclusion criteria: (1) Patients who had other malignancies. (2) Patients who had incomplete pathology examination or CT examination imaging data. (3) Patients whose first SPTP excision was not performed in our hospital. (4) Patients whose CT examination image was of poor quality and affected the detailed evaluation.

### Examination method

The 320-slice dynamic volume CT, known as Aquilion One, manufactured by Toshiba was used for the scanning. All patients underwent plain scanning for 25–30 s during the arterial phase, 40–45 s during the pancreatic parenchymal phase, 60–65 s during the portal vein phase and 110–120 s during the delayed phase. Scanning parameters: The tube voltage was 120 kV, the milliampere second was 150 mAs, the bulb tube rotation time was 0.5 s per revolution, the detector collimation was 100 × 0.5 mm, the data reconstruction thin-slice thickness was 1 mm, the slice gap was 0.8 mm, and the FOV was 350 mm×350 mm.

Target scanning was adopted during the pancreatic parenchymal phase: The tube voltage was 120 kV, the milliampere second was 250 mAs, the detector collimation was 320 × 0.5 mm, the data reconstruction thin-slice thickness was 0.5 mm, the slice gap was 0.25 mm, the FOV was 200 mm×200 mm, and other parameters were the same as those in the above scanning scheme. Contrast agent injection protocol: 60–100 mL Iopamidol (370 mg I/mL), a nonionic contrast agent, was injected using the double-syringe high-pressure injector, at a rate of 4.5–5.0 mL/s, followed by 20 mL 0.9% normal saline.

### CT image analysis

Two attending doctors with more than five years of experience in diagnosis of pancreatic tumors were responsible for independently reviewing the CT images for the tumor features, pancreatic and bile duct dilation, vascular invasion and metastasis, and other aspects. In case of disagreement, consensus was reached through consultation. For evaluation of tumor enhancement features, solid components of the tumor and adjacent normal pancreatic parenchyma were selected while calcified, vascular, bleeding, liquidated, and other such regions were avoided.

### Pathology examination

For postoperative tumor specimens, the surgical margin was observed, routine hematoxylin and eosin (H&E) staining and immunohistochemistry (IHC) detection were performed, and two senior pathologists confirmed the diagnosis.

### Observation indexes

(1) CT imaging features: tumor location, size, morphology, growth pattern, integrity of capsule, cystic or solid [solid (solid components > 70%), cystic-solid (solid components: 30–70%), cystic (solid components < 30%)], calcifications, plain scanning, and enhancement features. (2) Pathology examination: The surgical margin, tumor size, growth pattern, integrity of capsule (observed during the pancreatic parenchymal phase where target scanning was adopted), cystic or solid, and calcifications were observed. (3) Treatment and follow-up.

### Treatment and follow-up

After completion of preoperative investigations, the indicated surgery was performed according to the imaging features of the tumor and patients’ general conditions, keeping in mind the choice of the patients and their family members. Surgery was followed by postoperative pathology examination. We conducted outpatient and telephonic follow-up every three months for half a year post-surgery, and once every 6 months thereafter, so as to understand patients’ clinical symptoms and signs, and tumor recurrence or metastasis. The follow-up ended in May 2018.

### Statistical analysis

We used the SPSS19.0 statistical software for analysis. Measurement data in normal distribution are expressed as mean ± SD, and those in abnormal distribution are expressed as median. Measurement data were compared using Student’s t-test, and enumeration data were compared using Fisher’s exact test. *P* < 0.05 indicated statistically significant differences.

## Results

### Clinical features

The 27 patients with SPTP consisted of 77.8% of females and 22.2% of males, with a male-female ratio of about 1:3.5. There were statistical differences in the age of onset between females [(41.2 ± 13.9) years] and males [(33.1 ± 10.1) years] (t = -7.228, *P* < 0.05).

### CT examination

#### General CT findings of SPTP

**Fig. 1:**
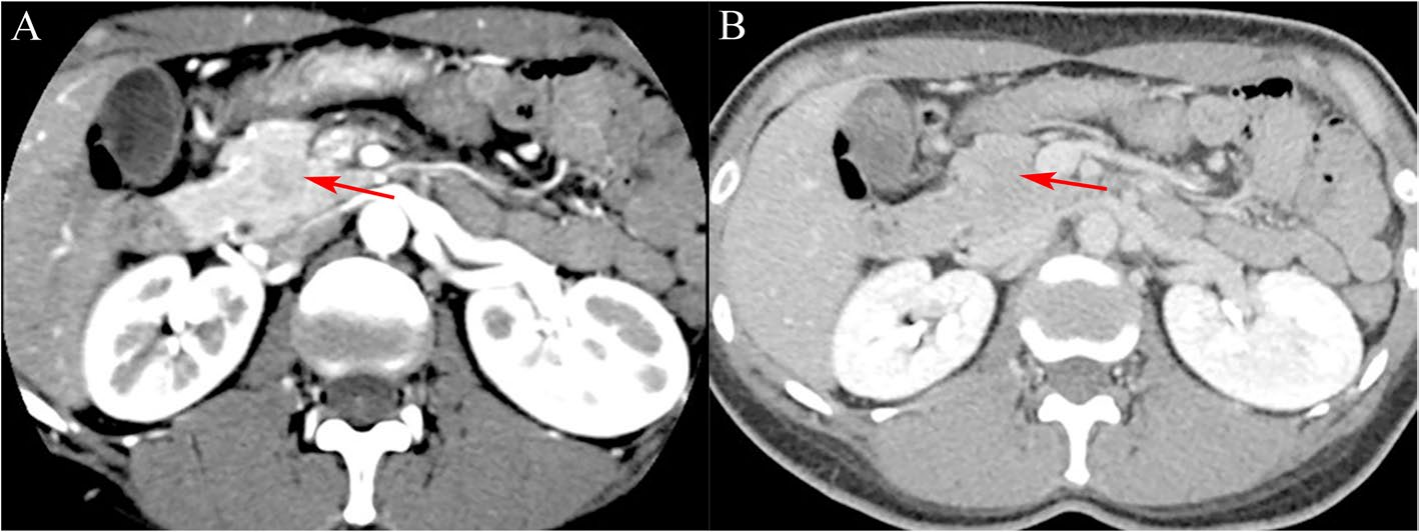
A 25-year-old male with a solid pseudopapillary tumor. CT target scanning shows that (**a**) the SPTP is located on the pancreatic head, and the capsule is complete and enhanced linearly (long arrow) during the pancreatic parenchymal phase; (**b**) the tumor is enhanced to the same degree as the pancreas and shows isoenhancement during the delayed phase (long arrow)

**Fig. 2:**
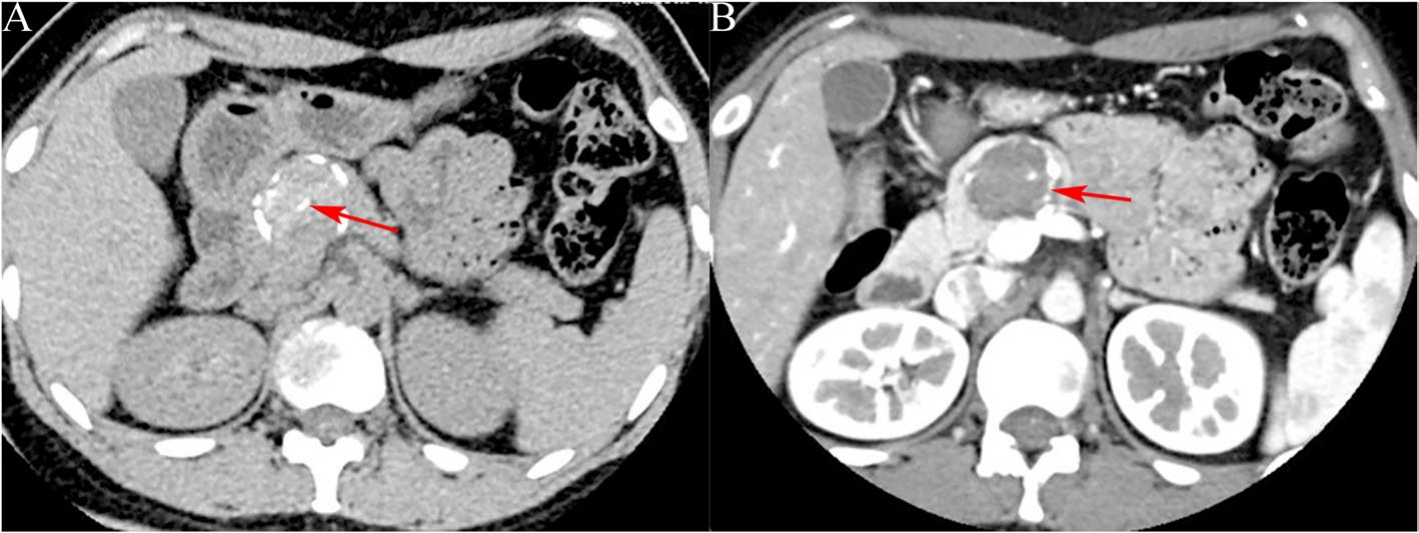
A 47-year-old female with a solid pseudopapillary tumor. **a** Plain CT scanning shows that the SPTP is located on the pancreatic head, with isodensity and central punctate calcifications (long arrow) and marginal semiarc calcifications of the tumor; **b** CT target scanning shows that the solid components in the tumor center are mildly enhanced (long arrow), to a lower degree than the surrounding normal pancreatic parenchyma during the pancreatic parenchymal phase

**Fig. 3:**
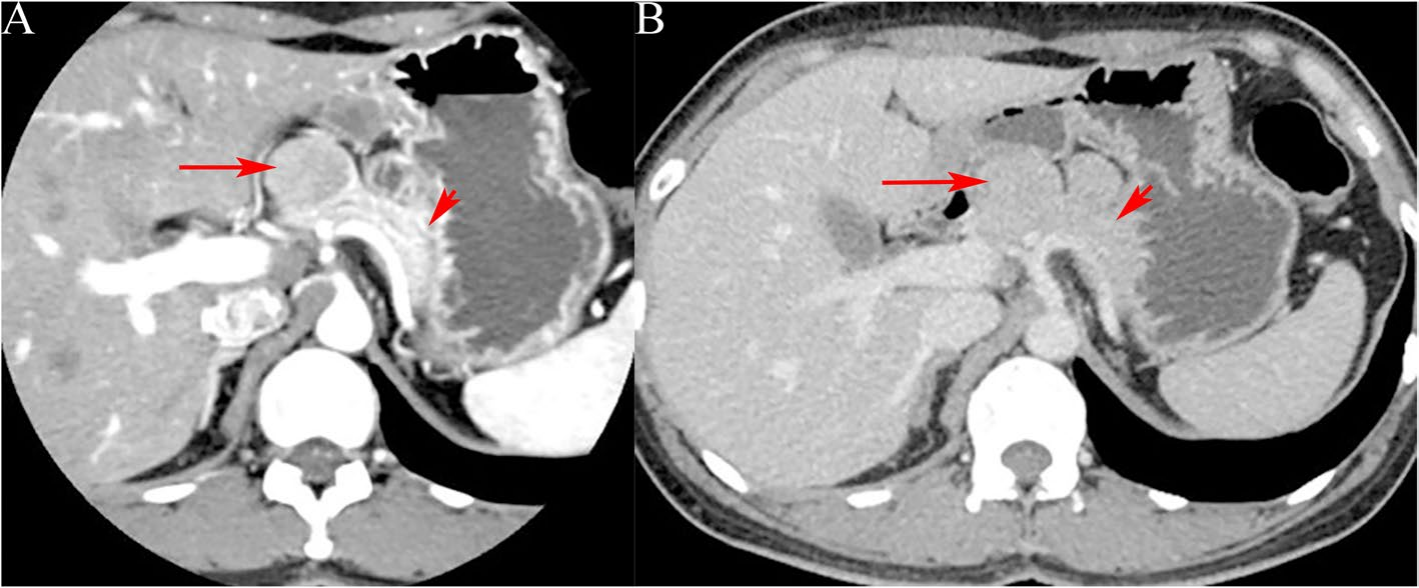
A 47-year-old female with solid pseudopapillary tumor. **a** CT target scanning shows that the SPTP is located on the pancreatic neck with a complete capsule and solid tumors, and the tumors (long arrow) are enhanced to a lower degree than the normal pancreatic parenchyma (short arrow) during the pancreatic parenchymal phase; **b** the tumors (long arrow) are enhanced to the same degree as the normal pancreatic parenchyma (short arrow) during the delayed phase

**Fig. 4:**
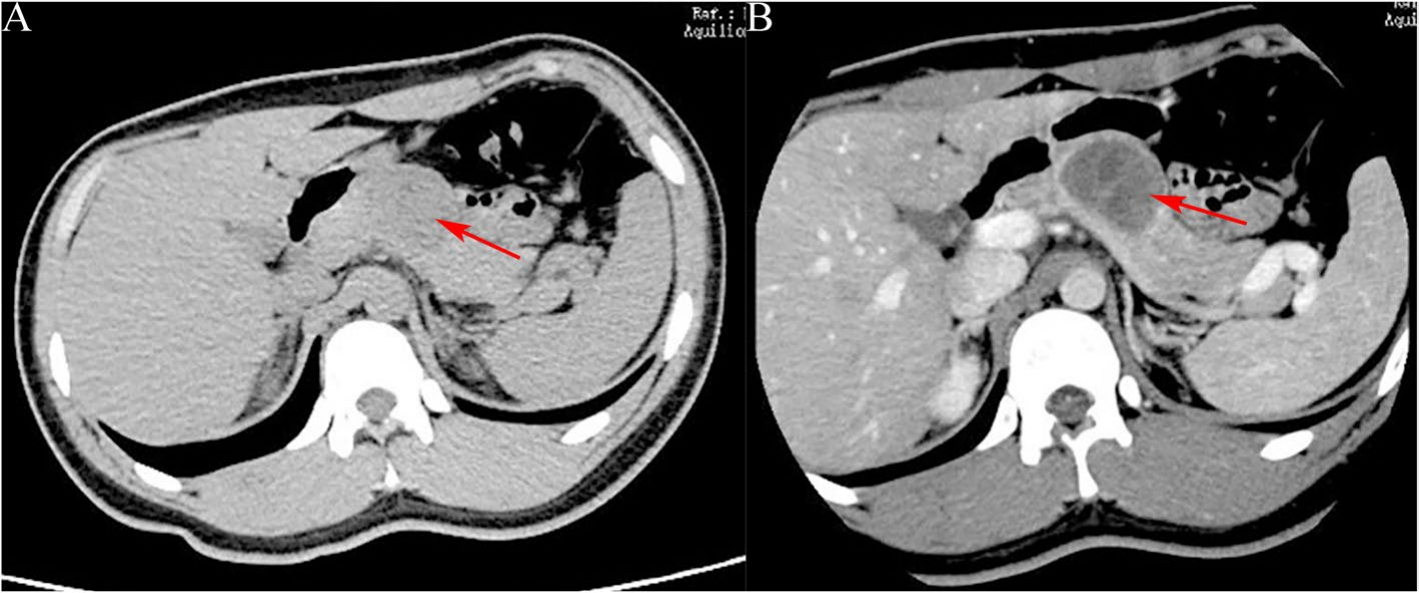
A 17-year-old male with solid pseudopapillary tumor. **a** Plain CT scanning shows a quasi-circular slightly lower density shadow of the pancreatic body (long arrow); **b** CT target scanning shows that the tumor capsule is complete and enhanced solid components (long arrow) are seen on the margin during the pancreatic parenchymal phase

**Fig. 5:**
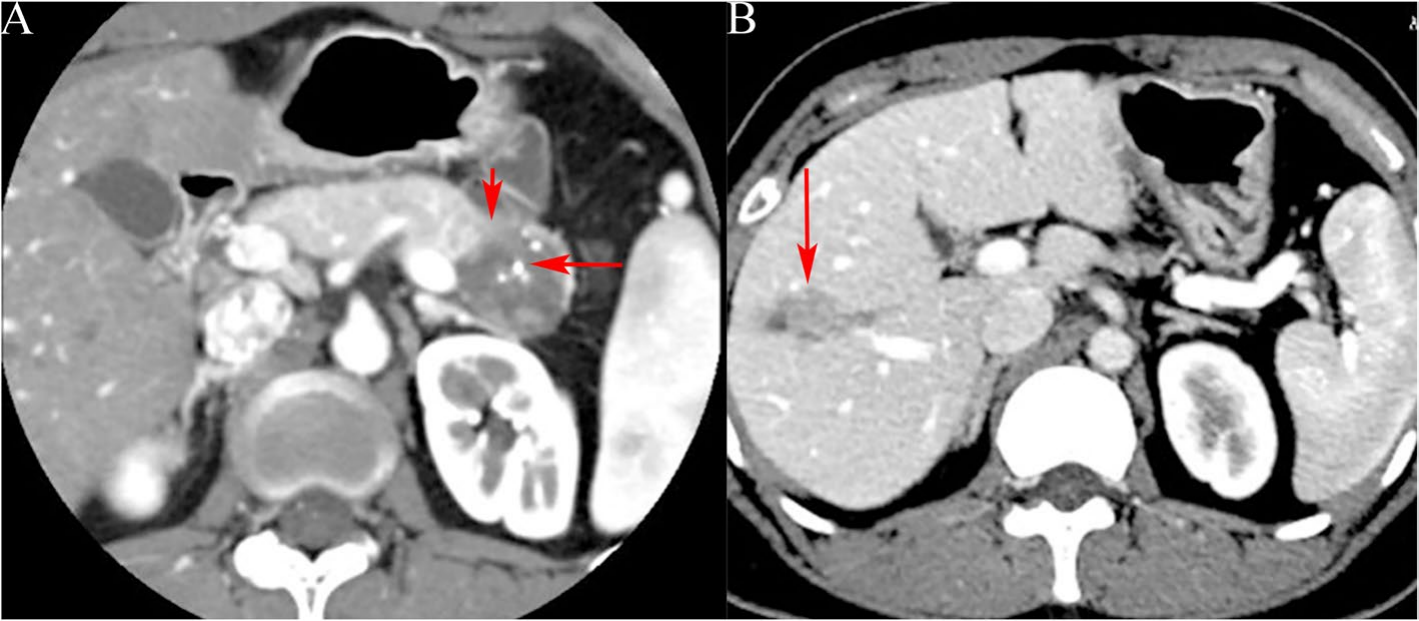
A 36-year-old female with solid pseudopapillary tumor. **a** CT target scanning shows that SPTP is located on the pancreatic tail and is quasi-circular, with scattered nodular calcifications (long arrow) inside and incomplete capsule (short arrow) during the pancreatic parenchymal phase; **b** Lesions of the right lobe of the liver (long arrow) are observed, which are relatively lowly enhanced during the portal vein phase, and the pathology results confirmed it as SPTP liver metastasis

**Fig. 6:**
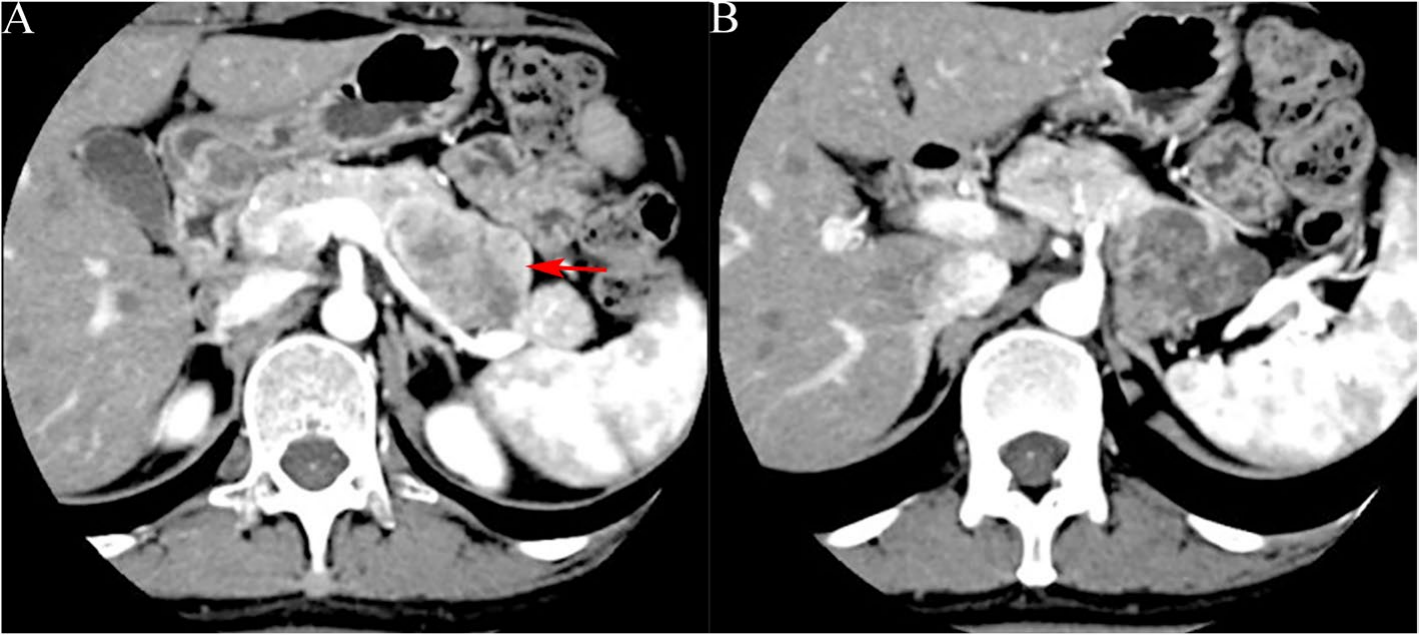
A 37-year-old male with solid pseudopapillary tumor. CT target scanning shows that (**a**) the tumor is located on the pancreatic body and tail and is triangle-like, the tumor capsule is complete and linearly enhanced obviously (long arrow) during the pancreatic parenchymal phase; (**b**) the lower margin of the tumor is lobulated and grows out of the pancreas during the pancreatic parenchymal phase

(1) Location: Pancreatic head in 9 (33.33%)cases, pancreatic neck in 2(7.41%)cases, and distal pancreas (pancreatic body and tail) in 16 (59.26%)cases. (2) Size: The diameter range was 2–14 cm, with the average diameter of (4.3 ± 2.6) cm. (3) Morphology: Quasi-circular tumors in 22(81.48%) cases (Figs. 1, 2, 3, 4 and 5), and lobular tumors in 5 (18.52%)cases (Fig. 6), with the diameter of lobular tumors in 5 cases being > 4.0 cm. (4) Growth pattern: The tumor center was inside the pancreas in 14(51.85%) cases, and outside the pancreas in 13(48.15%)cases. (5) Integrity of capsule: The capsule was complete in 22(81.48%) cases (Figs. 2, 3 and 6), and incomplete in 5(18.52%) cases (Figs. 3 and 6). (6) Cystic or solid: 9(33.33%) cases had solid (solid components > 70%) tumors (Figs. 1, 2, 3 and 5), 18(66.67%) cases had cystic-solid (solid components: 30–70%) tumors (Figs. 4 and 6), and there was no case of cystic (solid components < 30%) tumors [6]. Among 6(22.22%) cases with tumors < 3 cm, 4 (4/6)cases had solid tumors and 2 (2/6)cases had cystic-solid tumors; among 17 (62.96%)cases with tumors of 3–5 cm, 4 (4/17)cases had solid tumors and 13(13/17) cases had cystic-solid tumors; tumors > 5 cm in 4 (14.81%)cases were all cystic-solid. (7) Calcifications: The calcified margin or center of the tumor was observed in 16(59.26%) cases (Fig. 2). There were scattered punctate calcifications in 8 (8/16)cases, patchy calcifications in 4(4/16) cases, and marginal eggshell or short-arc calcifications in 4(4/16)cases. Calcification was not observed in 11(40.74%)cases. (8) Tumor plain scanning and enhancement features: Solid components of the tumor showed isodensity or slightly low-density. The solid components or cyst walls were mildly enhanced during the arterial phase, and were progressively enhanced during the pancreatic parenchymal phase, portal vein phase, and delayed phase, of which the degree of enhancement during each phase was lower than that of the normal pancreatic parenchyma in 24 (88.89%)cases, and the degree of enhancement became the same as that of the surrounding normal pancreatic parenchymal phase during the delayed phase in 3 (11.11%) cases (Fig. 3b); cystic components were not enhanced. The enhanced solid components were nebulous and mamillary against the low-density cystic necrosis region. (9) Pancreatic and bile duct dilation: Mild pancreatic duct dilation was observed in distal pancreas in only one(3.70%) case with an 8.8-cm pancreatic head tumor.

#### Vascular invasion and metastasis

The portal vein, superior mesenteric artery and vein, and splenic artery and vein travelled naturally, with no tumors encysting blood vessels and no vascular invasion; liver metastasis was observed in one case; no lymphatic metastasis was observed.

#### Comparison of CT signs between males and females

There were no statistical differences in the tumor location, morphology, growth pattern, integrity of capsule, cystic or solid, calcifications, and enhancement features between the male group and the female group (*P* > 0.05). In terms of the tumor size, the average diameter [(3.7 ± 0.6) cm] in the male group was smaller than that [(4.2 ± 2.2) cm] in the female group, and there were significant differences between the two groups (t = -4.869, *P* < 0.05) (Table 1).

**Table 1:** Comparison of CT signs between male and female patients (case)

Group	Number of cases	Location			Size (cm)		Morphology			Growth pattern	
		Pancreatic head	Pancreatic neck	Pancreatic body and tail			Quasi-circular	Lobulated		Inside pancreas	Outside pancreas
Male group	6	2	1	3	3.7±0.6		5	1		4	2
Female group	21	7	1	13	4.2±2.2		17	4		10	11
Total	27	9	2	16	4.3±2.6		22	5		14	13
Statistical value					t=-4.869						
*P* value		0.605			0.000		1.000			0.648	
Group	Number of cases	Integrity of capsule			Cystic or solid			Calcifications		Enhancement features	
		Complete	Incomplete		Solid	Cystic-solid	Cystic	Yes	No	Low enhancement	Isoenhancement
Male group	6	5	1		5	1	5	4	2	6	0
Female group	21	17	4		17	4	17	12	9	18	3
Total	27	22	5		22	5	22	16	11	24	3
Statistical value											
*P* value		1.000			0.628			1.000		1.000	

### Pathology examination results

#### Gross specimen

(1) Negative incisal margins of tumors were seen in 27 cases, all of which were R0 excision. (2) Morphological features of gross tumor: The tumor was quasi-circular in 22 cases, and lobular or irregular in 5 cases. (3) Integrity of capsule: The capsule was complete in 24 cases, and incomplete in 3 cases. (4) Tumors were solid in 10 cases, cystic-solid in 17 cases, and cystic in 0 case. (5) Calcifications: Calcifications were observed in 16 cases, and no calcifications were observed in 11 cases.

#### H&E staining detection

In terms of the morphology, the tumors were nestlike and mamillary under the microscope, with nerve invasion in 2 cases, and necrosis in 17 cases, and no vessel carcinoma embolus or lymphatic metastasis was observed. Tissue with histological morphology similar to that in the pancreatic tumor was seen in the hepatic tumor in one case.

#### IHC test

The IHC test showed that the nuclear positive expression rate of lymphatic enhancer factor-1 (LEF-1) was 100% (27/27 cases), and the positive expression rate of β-catenin was 100% (27/27 cases).

### Comparison of CT signs and pathology

Determination of integrity of capsule: Capsules were shown incomplete in CT findings and yet complete in pathology in 2 cases, suggesting a CT accuracy rate of 92.6%. Determination of cystic or solid: Tumors were shown cystic-solid in CT findings and yet solid in pathology in 1 case, suggesting a CT accuracy rate of 96.3%.

Determination of metastasis: The CT findings of liver metastasis were consistent with the pathology results in 1 case. Both CT and pathology findings showed no lymphatic metastasis.

### Treatment and follow-up

Three cases underwent radical pancreaticoduodenectomy, 5 cases underwent pylorus-preserving pancreaticoduodenectomy, 10 cases underwent distal pancreatectomy + splenectomy (including 5 cases by laparotomy, and 5 cases by laparoscopy), 4 cases underwent distal pancreatectomy, 4 cases underwent segmental pancreatectomy, and 1 case underwent distal pancreatectomy + splenectomy + special hepatic segmentectomy + partial jejunectomy + cholecystectomy.

Pancreatic fistula (grade B) was found in 2 cases and delayed gastric emptying in 1 case post-surgery. After treatment, these complications were resolved, with no death, and the one-year survival rate reached 100%. All 27 cases were followed up for 2–20 months, with a median time of 14 months. No obvious recurrence or metastasis was observed during follow-up.

## Discussion

### Clinical features

The theory that SPTP may originate from germinal ridge-ovary primordia-associated cells during embryogenesis can also explain the higher diagnosis of the disease in women [7]. SPTP is of unknown pathogenesis and low malignancy potential, and yet is rarely metastatic and has good prognosis after surgical excision [8]. Although rare, SPTP is the most common pathological type representing 72.4% of pancreatectomy cases in patients under 40 years of age [9]. In this study, patients developed the disease at an average age of 39 years, slightly higher than the average of 34 years reported by Hu et al. [10] and yet significantly higher than the average age of 29 years reported by Zhan et al. [11]. In addition, 66.7% (18/27 cases, including 14 females and 4 males) of patients were aged under 40 years, which was consistent with the higher diagnosis in young women [12]. The male-female ratio in this study was 1:3.5, comparable with the 1:5.9 and 1:10 reported by other scholars [7, 11].

SPTP has atypical clinical manifestations such as abdominal pain or discomfort, lumbar and back pain, and other tumor compression symptoms. Half of the patients in this study were diagnosed via physical examination. Since the tumor tissue is soft enough not to oppress the pancreatic and bile duct and cause obstructive symptoms, only one case developed secondary pancreatic duct dilation in the giant pancreatic head tumors, but with no bile duct dilation and obstructive jaundice symptoms.

Because CT examination has the characteristics of wide application, rapidity, and clear anatomical display, it is crucial for the detection of both symptomatic and asymptomatic SPTP tumors, but it is very easy to miss the diagnosis when the difference in density between the tumor and the surrounding normal pancreatic parenchyma is not obvious. Among the 10 cases in this group in which no calcification was seen, 4 cases of tumors smaller than 4 cm were isointense on CT plain scanning, and it was difficult to detect the lesions by CT plain scanning alone. Enhanced CT with target scanning can help to detect these tumors.

The iterative reconstruction (IR) algorithm is a commonly used reconstruction algorithm for CT images, which can reduce the radiation dose. Target scanning refers to the method of scanning after local magnification of the area of interest, which can significantly improve the spatial resolution of the image, more realistically respond to the density and anatomical relationship of the tissue, and improve the clarity of small lesions, and is mainly used for scanning lung nodules, and has not yet been reported to be applied to the pancreas. In this group of cases, target scanning was used in the parenchymal phase of the pancreas, and iterative reconstruction was used in the plain scanning, arterial phase, portal phase and delayed phase to effectively reduce the radiation dose.

### CT examination

#### Advantage of CT iterative reconstruction combined with target scanning

Thanks to its widespread application, rapidity, and clarity in detailing anatomical features, the CT examination is an essential tool for the detection of both symptomatic and asymptomatic SPTP, with the only exception being that the tumors are easily missed when they are of insignificant density differences from the surrounding normal pancreatic parenchyma. Among 10 cases without calcifications in this study, 4 cases with tumors < 4 cm showed isodensity in the plain CT scanning, suggesting that the lesions were difficult to detect based only on plain CT scanning. However, they could be detected by the enhanced CT of iterative reconstruction combined with target scanning.

Common reconstruction algorithms of CT images mainly include the iterative reconstruction (IR) algorithm [12]. Arapakis et al. [13] reported an effective dose reduction and clearer images using dose iterative reconstruction technique in abnormal CT scanning. Target scanning means scanning the area of interest that is locally magnified, which can significantly improve the spatial resolution of images, reflect the density and anatomical relationships of tissues more accurately, and improve the definition of small lesions. In this study, we used target scanning during the pancreatic parenchymal phase, and we used iterative reconstruction technique for plain scanning, and during the arterial phase, portal vein phase, and delayed phase to effectively reduce the radiation dose.

### General CT findings of SPTP

(1) Location: Although SPTP can occur in any part of the pancreas, it is most commonly diagnosed in the pancreatic tail. In this study, the distal pancreas accounted for 59.2% (16/27 cases), which was close to the 57.1% reported by Rai et al. [5].

(2) Size: The average size of the tumor was 4.3 cm, close to the 4.4 cm reported by Chen et al. [9] and significantly smaller than the 7 cm reported by Zhan et al. [11] In this study, the tumor in male patients was smaller than that in female patients, and the gender difference was statistically significant.

(3) Morphology: Most of the tumors were quasi-circular, and a few were lobulated. In this study, the 5 cases of lobulated tumors were larger than 4 cm in diameter, suggesting that the tumors were in multicentric expansive growth when they were large.

(4) Growth pattern: The tumor center can be located inside the pancreas or protrude outward; tumors in exocentric growth often need to be distinguished from lymph node tumors or retroperitoneal neurogenic tumors. The enhanced scanning showed a “flared” change in the boundary between the tumor and the pancreas (Figs. 2 and 6). This often suggests that the tumor is from the pancreas, which is helpful for the localization diagnosis of the tumor.

(5) Capsule: A tumor with a complete capsule appears on the CT image as a clear capsule and has a clear boundary with the surrounding tissue; when the tumor perforates the capsule, an incomplete tumor capsule and an unclear boundary show on the CT image. Enhanced scanning showed that = the capsule is obviously enhanced (Fig. 6a). Wang et al. [13] found in their study that tumors larger than 6 cm were related to capsular invasion. However, in this study, 4 of the 5 cases of tumors with incomplete capsules were smaller than 6 cm (except for only 1 case where it was larger than 6 cm), which is inconsistent with literature and this requires further study with large-sample data.

(6) Cystic or solid and CT findings: Due to the heterogeneity of the tumor, there can be bleeding, necrosis, or cystic degeneration in the tissue, which appear as iso- and high-density, high- and low-density, and low-density changes on the plain CT scanning. In this study, 67.7% (4/6 cases) of the tumors that were smaller than 3 cm were solid, and 100% (4/4 cases) of those larger than 5 cm were cystic-solid. This may be because bleeding, necrosis, or cystic degeneration occur with the growth of the tumor while none of them are found in small tumors. Miao et al. [14] found that when cystic and solid components were in similar proportions, the solid components were flakey and were obviously enhanced after enhancement, showing “floating cloud signs” against the low-density cystic components; when cystic and solid components were alternatively distributed, the cystic wall nodule showed a “pseudopapillary structure”; when the solid components dominated, the cystic components showed a small subcapsular circular structure.

While the CT showed that the solid components were mamillary or flocculent located on the periphery, the pathology results showed that a solid region consisted of fibrous blood vessels and nestlike or patchy tumor cells, as well as a pseudopapillary region consisting of tumor cells around the connective tissue such as thin blood vessels; the centric cystic components consisted of bleeding and degenerative tissue containing fibrin components [15]. This explains, pathologically, why the solid components are progressively enhanced on the CT enhanced scanning while the cystic components are not [16–19]. In this study, the 24 cases of tumors were progressively enhanced to a degree lower than the surrounding normal pancreatic parenchyma, and 3 cases were progressively enhanced but were isoenhanced during the delayed phase.

(7) Calcifications: Central scattered punctate calcifications and marginal semiarc eggshell calcifications may occur in the tumor, with septal calcifications in some tumors [20]. The calcification rate of 59.3% (16/27 cases) in this study was far higher than the 35.3% (12/34 cases) reported by Li et al., [21] but this may be related to the selection of the cases. SPTP should be considered first when the solid components of the tumor are progressively enhanced, with calcifications observed in the lesions.

#### Vascular invasion and Metastasis

Vascular invasion and metastasis: Although SPTP is classified as a malignancy, it does not invade the arteries and veins around the tumor, and does not cause vascular stenosis or truncation, which is also a feature different from the duct adenocarcinoma. SPTP may metastasize to the regional lymph nodes, mesentery, omentum majus, and peritoneal metastasis, while liver metastasis the most common. In this study, liver metastasis occurred in only 3.7% (1/27 cases) of patients, and no other metastases were found.

#### Comparison of CT signs between male and female patients

There were statistically significant differences between the male group and the female group only with respect to the tumor size. The tumors in the male group were slightly smaller than those in the female group; 83.3% (5/6 cases) of male patients who showed no obvious clinical symptoms were incidentally detected with pancreatic lesions during the physical examination, and 16.7% (1/6 cases) visited doctors due to abnormal pain for two weeks. This suggests that male patients pay more attention to physical examination and are detected with tumors earlier than female patients. Therefore, advocating physical examination is of significance in the detection of tumors such as SPTP that have slow growth and no typical clinical symptoms.

### Pathology examination results

Microscopically, it was found that the solid components of SPTP mainly consist of the solid patchy region, the pseudopapillary region, and the transition region of the two; the tumor cells were quasi-circular and middle-sized; the cells were less atypical, and the tumor cells could form the characteristic dendroid pseudopapilla around the blood vessels. The cystic region was mainly composed of bleeding, necrosis, and mucoid degeneration [22]. Hu et al. [10] conducted the IHC test on 132 SPTP patients and found that 98.5% (130/132 cases) developed nuclear positive expression of lymphatic enhancer factor-1 (LEF-1), while no LEF-1 expression was found in other pancreatic tumors and surrounding normal pancreatic tissue, with a specificity of 100%. In this study, the positive expression rates of LEF-1 and β-catenin in SPTP were 100%, which is consistent with literature.

### Comparison of CT signs and pathology

With pathology examination as the golden standard, CT examination had an accuracy of 92.6% and 96.3% in the evaluation of the integrity of pancreatic capsule and the cystic or solid tumors. The two cases whose CT findings showed incomplete capsules may be because the enhanced scanning failed to completely show the capsule, due to which the boundary between the local part of the tumor and the pancreatic parenchyma was unclear and thus led to misjudgment. For one case with cystic-solid tumors, the plain CT scanning showed slightly low-density, and the target scanning during the pancreatic parenchymal phase showed relatively low enhancement, while the pathology results showed solid tumors. The reason may be that the mildly enhanced part of the solid region of the tumor was mistaken as there being no enhancement.

### Treatment and follow-up

With plain CT scanning and enhanced scanning, doctors can accurately locate the tumor, and evaluate its adhesion and invasion to the surrounding tissue and blood vessels, thereby being able to select the most appropriate surgical plan. Patients with pancreatic head tumors may undergo radical gastroduodenectomy, or pylorus-preserving pancreaticoduodenectomy based on the relationship between the tumor and adjacent organs, thus helping improve the quality of life post-surgery. For smaller tumors on the pancreatic body, segmental pancreatectomy is an option to reduce postoperative complications. Patients had satisfactory prognosis, with few recurrences or metastases [23, 24].

CT features are mainly used to distinguish between SPTP and pancreatic neuroendocrine tumors (pNETs). The degree of enhancement of SPTP is generally lower, while that of the latter is significantly higher during the arterial phase or portal vein phase, compared with the surrounding normal pancreatic parenchyma. However, it is difficult to distinguish between the above two when SPTP is as isoenhanced as the pancreatic parenchyma. In this study, 3 cases showed isoenhancement during the delayed phase, including 1 case that was misdiagnosed with pNETs based on preoperative CT.

## Conclusion

In conclusion, we found that SPTP has specific clinical and CT features. This disease is more common in young females, with a low level of tumor markers. CT iterative reconstruction technology combined with targeted scanning technology showed a cystic-solid structure, where calcifications were visible and the capsule was clear and complete, and enhanced scanning showed that the solid components were progressively enhanced. Notably, SPTP can be considered when there is no pancreatic and bile duct dilation. In our study, we found that iterative reconstruction combined with target scanning clearly displayed the CT features of the tumor and reflected the pathological changes of the tumor.

## Data Availability

The datasets used and/or analysed during the current study available from the corresponding author on reasonable request. We declared that materials described in the manuscript, including all relevant raw data, will be freely available to any scientist wishing to use them for non-commercial purposes, without breaching participant confidentiality.
